# The Effect of Tomatine on Gene Expression and Cell Monolayer Integrity in Caco-2

**DOI:** 10.3390/molecules23030644

**Published:** 2018-03-13

**Authors:** Mattia P. Arena, Coen Govers, Concetta Lotti, Luigi Ricciardi, Harry J. Wichers, Jurriaan J. Mes

**Affiliations:** 1 Department of the Sciences of Agriculture, Food and Environment, University of Foggia, Via Napoli 25, 71122 Foggia, Italy; mattiapia.arena@unifg.it; 2 Wageningen Food & Biobased Research, Wageningen University and Research, Bornse Weilanden 9, P.O. Box 17, 6700 AA Wageningen, The Netherlands; coen.govers@wur.nl (C.G.); harry.wichers@wur.nl (H.J.W.); jurriaan.mes@wur.nl (J.J.M.); 3 Department of Soil, Plant and Food Sciences, Plant Genetics and Breeding Unit; Via Amendola 165/A, 70125 Bari, Italy; luigi.ricciardi@uniba.it; 4 Laboratory of Food Chemistry, Wageningen University and Research Centre, Bomenweg 2, P.O. Box 8129, 6700 EV Wageningen, The Netherlands

**Keywords:** tomatine, glycoalkaloid, immune system, gut, gene expression, qPCR

## Abstract

More understanding of the risk-benefit effect of the glycoalkaloid tomatine is required to be able to estimate the role it might play in our diet. In this work, we focused on effects towards intestinal epithelial cells based on a Caco-2 model in order to analyze the influence on the cell monolayer integrity and on the expression levels of genes involved in cholesterol/sterol biosynthesis (LDLR), lipid metabolism (NR2F2), glucose and amino acid uptake (SGLT1, PAT1), cell cycle (PCNA, CDKN1A), apoptosis (CASP-3, BMF, KLF6), tight junctions (CLDN4, OCLN2) and cytokine-mediated signaling (IL-8, IL1β, TSLP, TNF-α). Furthermore, since the bioactivity of the compound might vary in the presence of a food matrix and following digestion, the influence of both pure tomatine and in vitro digested tomatine with and without tomato fruit matrix was studied. The obtained results suggested that concentrations <20 µg/mL of tomatine, either undigested or in vitro digested, do not compromise the viability of Caco-2 cells and stimulate cytokine expression. This effect of tomatine, in vitro digested tomatine or in vitro digested tomatine with tomato matrix differs slightly, probably due to variations of bioactivity or bioavailability of the tomatine. The results lead to the hypothesis that tomatine acts as hormetic compound that can induce beneficial or risk toxic effects whether used in low or high dose.

## 1. Introduction

Glycoalkaloids are steroidal secondary metabolites present in plants of the *Solanum* genus, including potato (*Solanum tuberosum* L.) and tomato (*Solanum lycopersicum* L.), which play a role in plant resistance against fungi, bacteria, virus and insects [1,2].

**Figure 1:**
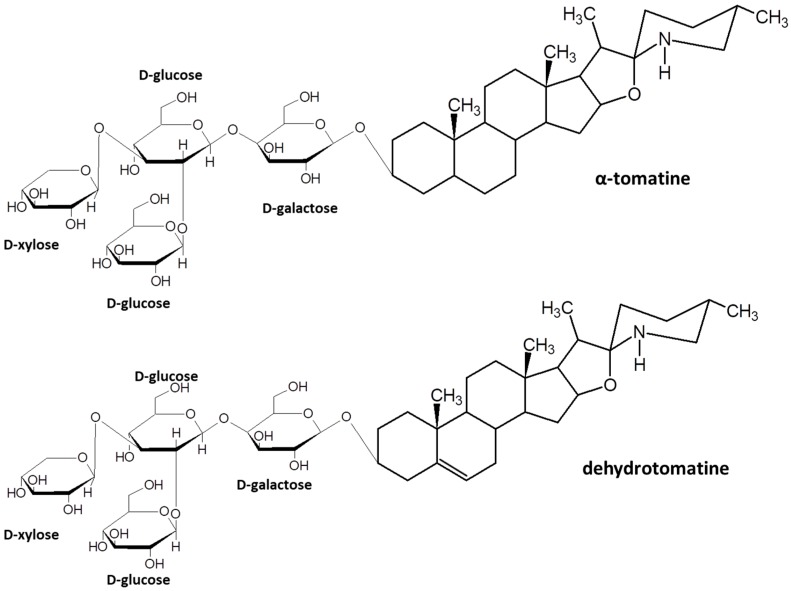
Chemical structures of α-tomatine and dehydrotomatine drawn using ACD/ChemSketch.

Tomatine is a mixture of two glycoalkaloids called α-tomatine and dehydrotomatine generally found in all parts of tomato plants (Figure 1). During the maturation stages, tomatine is degraded in the fruit: immature green tomatoes can contain up to 500 mg/kg of fresh weight (FW), while red ripe tomatoes up to 5 mg/kg (FW) [3]. More in detail, glycoalkaloid content, whose biosynthesis involves gene cluster coding for glycosyltransferases, dehydrogenase and reductase, is inversely proportional to the weight and the diameter of the fruit, and it is not correlated to the position of the fruit on the stem. Moreover, the amount of glycoalkaloid in the fruits, as well as in the other parts of tomato plants, is highly variable and depends on cultivar and growing conditions [4]. In several tomato biotypes, which show high content in glycoalkaloids, a mutation in GAME1 gene involved in the degradation of tomatine during the maturation stages was identified [5,6]. Tomato fruits with low glycoalkaloids content were commonly preferred as tomatine has been considered potentially toxic based on the known toxicity of potato glycoalkaloids in humans [7]. However, little is known about the real safety profile, the bioactivity, the availability, the metabolism, and the pharmacokinetics of tomatine. α-Tomatine and dehydrotomatine have both been shown to be competitive inhibitors of bovine and human acetylcholinesterase, although less effective than potato glycoalkaloids [8]. Besides it is shown that these compounds have the ability to bind cholesterol and other 3β-hydroxysterols such as cholestanol and lanosterol, consequently damaging cell membrane integrity [9]. In contrast to potato glycoalkaloids, no toxic effects in humans have been reported for tomato alkaloids in the literature [10,11]. Recent research on tomatine has dealt with the pharmacological and nutritional roles that the tomato glycoalkaloids may play in the human diet. Interestingly, tomatine seems to exhibit anticancer, chemopreventive, anticholesterol, anti-inflammatory, antipyretic, antifungal and antibacterial properties [8,9]. In vitro studies showed that tomatine interferes with active transport by increasing the permeability of human mucosal epithelial cell membranes and altering the membrane potential [11,12]. Furthermore, some studies, using aggregate formulations containing α-tomatine, supported the immune-potentiating properties of tomato glycoalkaloid [13,14,15]. Moreover, tomatine has been shown to inhibit growth in a number of human cancer cell lines [16,17,18]. An in vivo study of the glycoalkaloids effects on rats showed that dietary α-tomatine binds cholesterol, resulting in an [19,20]. In addition, tomatidine inhibits COX-2 expression involved in inflammatory processes [21].

The main aim of this work was to gain insight on the effects of tomatine on the human intestinal enterocyte cell line Caco-2 in order to develop a model to analyze the dose-dependent risk-benefit effects towards these cells that are of major importance to maintain homeostasis in the intestine. Furthermore, since the bioavailability and bioactivity of the compounds might vary in the presence of food matrix and following the food digestion, we studied the influence of the pure tomatine but we also considered in vitro digested tomatine with and without tomato fruit matrix.

## 2. Results and Discussion

### 2.1. HPLC Analysis Confirmed the Presence of Tomatine after In Vitro Digestion

To the best of our knowledge, glycoalkaloids are considered toxic secondary metabolites and the catabolism of these compounds by humans is not well elucidated. In order to understand whether tomato glycoalkaloid (α-tomatine and dehydrotomatine) content could be altered after ingestion, in vitro digestion assays were carried out. Thus, pure tomatine standard (A), tomato fruits spiked with pure tomatine standard (T) and tomato fruits (T_c_) were exposed to different abiotic stresses mimicking the gastric and intestinal environments, such as acidic conditions, exposure to enzymes (pepsin and pancreatin) and the presence of bile salts. Results indicated that both α-tomatine and dehydrotomatine were clearly detectable in all samples after in vitro digestion, except in tomato fruits (T_c_) where both glycoalkaloids were not observable. The percentages of α-tomatine found in in vitro digested tomatine spiked to tomatoes (T) and in vitro digested pure tomatine (A) were comparable (28.9 ± 10.9 and 26.5 ± 7.5, respectively). Interestingly, the percentage of dehydrotomatine observed in the samples of in vitro digested tomatine spiked into tomatoes (T) (53.5 ± 18.1) was higher than that detected in the samples of in vitro digested pure tomatine (A) (26.0 ± 6.2) (data not shown). These findings suggested the hypothesis that α-tomatine and dehydrotomatine might be affected in different ways in the samples where tomato fruits are added (in vitro digestion with tomato (T)) respect to samples were tomato fruits were not added (in vitro digested pure tomatine and tomatine standard), probably due to compounds in tomato fruits which could interfere with and hinder the digestion of glycoalkaloids [22,23]. Supposedly, as polysaccharides have been shown to have a protective effect, we can hypothesize that pectins present in tomato fruits can create a mesh that could interfere with the availability of tomatine and, at the same time, could protect the tomatine from abiotic stresses during the gastrointestinal tract transit [24,25].

### 2.2. Caco-2 Monolayer Integrity is Influenced by Tomatine

Pure tomatine (P), in vitro digested tomatine with tomato matrix (T) and in vitro digested tomatine without tomato matrix (A) were incubated with Caco-2 cells in order to evaluate the intestinal epithelial electrical resistance (TEER), which is a measure of the flow of charge and reflects the paracellular permeability of the cell monolayer. Reducing TEER value could indicate a widening of tight junctions and a consequent increase in the paracellular transport of molecules and ions, but could also be caused by cell death.

**Figure 2:**
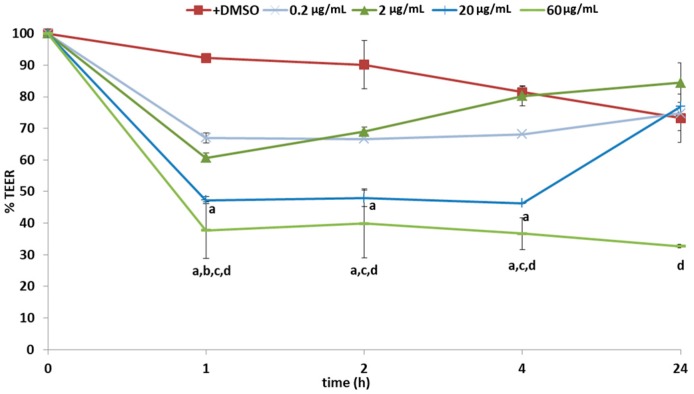
Effects of pure tomatine (P) on Caco-2 TEER values expressed as % relative to the control (DMEM + DMSO without tomatine). Values represent mean ± standard deviation of three different biological replications and two technical repetitions. Different superscript letters indicate statistically significant differences (*p* < 0.05) in adhesion as assessed by one-way ANOVA test.

Firstly, the influence of 12 concentrations of pure tomatine (P) (0.2–200 µg/mL corresponding approximately to 0.1–100 mg/kg (FW) of tomato), were tested on Caco-2 cells. Figure 2 shows the results for the exposure to 0.2, 2, 20 and 60 µg/mL tomatine. The effect of tomatine (P) occurs within the first hour of exposure with significant reduction of TEER values, ranging from 21% to 74%, in a concentration-dependent manner. Subsequently, an increase in TEER after 24 h of exposure at concentration up to 20 µg/mL of pure tomatine was observed. Conversely, concentrations >20 µg/mL significantly reduced TEER values to below 35% and apparently beyond the point of recovery. The results indicated that Caco-2 monolayer integrity is temporally reduced by low concentrations and permanently by high concentrations of pure tomatine (P).

**Figure 3:**
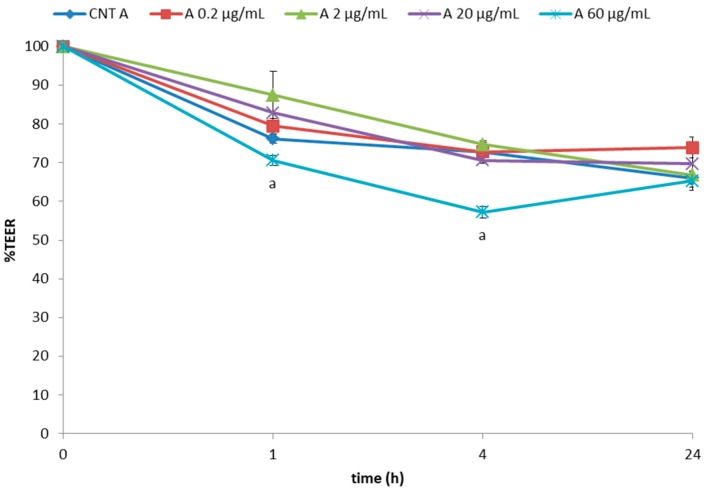
Effects of in vitro digested tomatine (A) on Caco-2 TEER values expressed as % relative to the control (AC). Values represent mean ± standard deviation of three different biological replications and two technical repetitions. Different superscript letters indicate statistically significant differences (*p* < 0.05) in adhesion as assessed by one-way ANOVA test.

**Figure 4:**
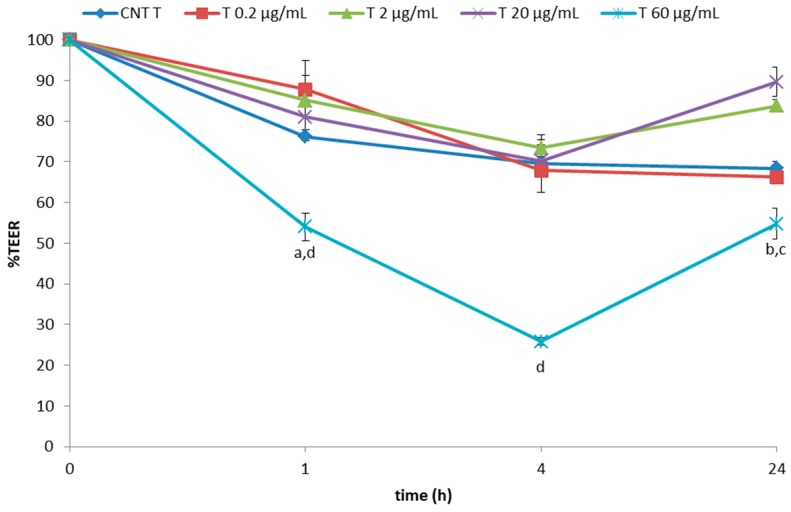
Effects of in vitro digested tomatine spiked to tomato matrix (T) on Caco-2 TEER values expressed as % relative to the control (TC). Values represent mean ± standard deviation of three different biological replications and two technical repetitions. Different superscript letters indicate statistically significant differences (*p* < 0.05) in adhesion as assessed by one-way ANOVA test.

On the basis of the above-mentioned results, four concentrations were chosen, i.e., 0.2 and 2 µg/mL (which did not decrease the TEER value below 74% over 24 h of exposure), 20 µg/mL (showing an effect on TEER that can still be recovered during a 24 h exposure), and 60 µg/mL (slightly higher than the limit potentially affecting the integrity of the cell monolayer), in order to study the effect of in vitro digestion and/or the presence of the tomato matrix on TEER lowering effect of tomatine. Overall, in vitro digested tomatine, both without (A) and with tomato red fruits (T), counteracted the TEER reduction as obtained using pure tomatine (P) (Figure 3 and Figure 4). This effect was especially corroborated by a milder effect using the high concentration of 60 µg/mL.

This counteracting effect of in vitro digestion might be due to the influence on tomatine stability due to pH changes, pepsinase and pancrease digestion, and bile salts [26]. Besides that, tomato fruits might interfere with the bioavailability of tomatine during the in vitro digestion [22] resulting in a lower impact on cell integrity.

### 2.3. Gene Expression Levels are Influenced by Tomatine

**Figure 5:**
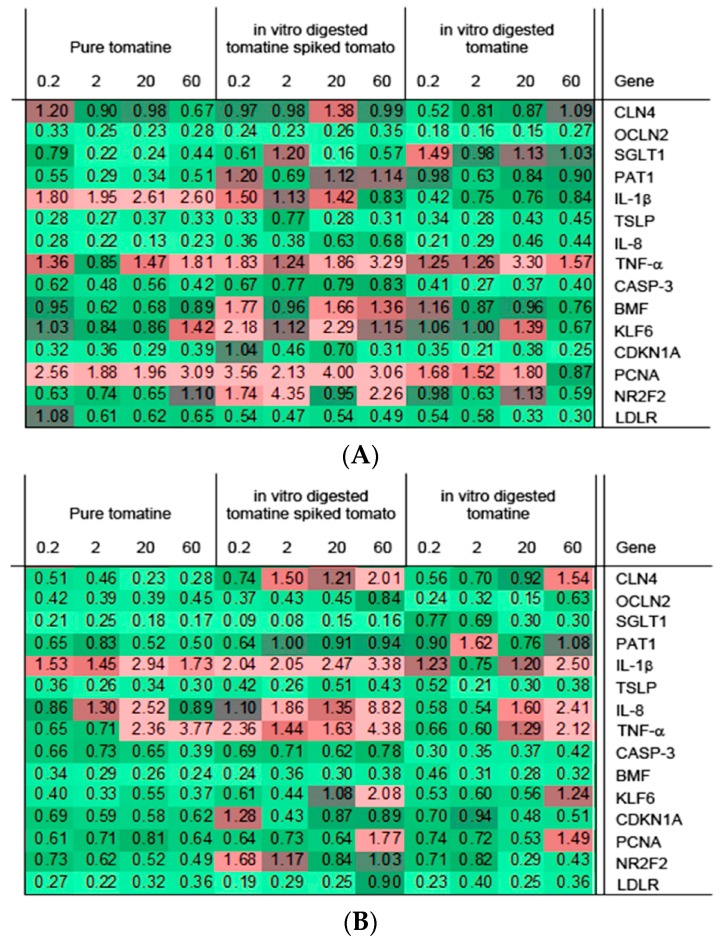
Relative gene expressions (RGE) resulted after 1 h (**A**) and 4 h (**B**) of exposure to pure tomatine, in vitro digested tomatine spiked to tomato matrix and in vitro digested tomatine treatments. All values are in comparison with the values of the control without tomatine (Pc).

Based on literature for other glycoalkaloids [27], it was hypothesized that the biological functions of the intestinal cells could be changed due to exposure to tomatine. That is why it was decided to study the effects of tomatine on biological pathways using specific gene expression markers: cholesterol/sterol biosynthesis (LDLR), lipid metabolism (NR2F2), glucose and amino acid uptake (SGLT1, PAT1), cell cycle (PCNA, CDKN1A), apoptosis (CASP-3, BMF, KLF6), tight junctions (CLDN4, OCLN2) and cytokine-mediated signaling (IL-8, IL1β, TSLP, TNF-α). The expression was determined by qPCR and the gene expression levels are shown in the Figure 5.

Firstly, we investigated whether the treatments induced more general toxic effects, which often include effects towards cell cycle and induction of apoptosis. Regarding this aspect, the cyclin-dependent kinase inhibitor 1A (CDKN1A) was not changed by the treatments (P, T, and A) within 1 h of exposure, while the transcription level of PCNA gene was increased. Based on observed effect of tomatine towards Caco-2 cells monolayer integrity, it could be expected that expression of claudin-4 (CLDN4) and occludin-2 (OCLN2) genes, coding for the tight junction (TJ) proteins which play a crucial role in the maintaining of the intestinal barrier integrity [28], would be effected. CLDN4 expression was reduced significantly in a concentration-dependent manner over all treatments of (P) after 4 h, and therefore correlated to TEER decrease. Conversely, using the in vitro digested tomatine (A) and tomatine with tomato matrix (T), expression analysis shows an up-regulation of CLDN4 at concentrations of 60 µg/mL after 4 h of exposure. This up-regulation of CLDN4 for intestinal cells with low TEER values have been observed before and probably can be seen as an attempt of the cells to restore the integrity of the tissue [29,30]. With respect to OCLN2 gene, the expression of transcriptional level was reduced significantly for almost all concentrations of (P), (A) and (T) treatments. Occludin proteins have been linked to the regulations of intermembrane and paracellular diffusion of small molecules [31]. One of the possible mechanisms to explain membrane disruption by glycoalkaloids involves the complexation with membrane cholesterol [8]. To study effect on the cholesterol LDLR and NR2F2 genes, that are involved in cholesterol and lipid biosynthesis, were selected as these genes showed a change in expression when Caco-2 cells were exposed to potato glycoalkaloids [27]. In our study, LDLR gene expression was reduced over all tomatine treatments, not in line with what have been found for potatoes glycoalkaloids. Instead NR2F2 gene was not subjected to significant changes respect to the control, with the exceptions of higher concentrations (20 and 60 µg/mL) for long exposure with (P) and (A) which resulted in a down regulations of expression. Down-regulation of NRF2 was also observed in Caco-2 upon exposure to potato glycoalkaloids [27]. Our data indicate that tomato glycoalkaloids cause less disturbance of the cellular cholesterol homeostasis than potato glycoalkaloids. We suggest as possible explanation of the NR2F2 gene increment with the treatments (T), an effect due to the presence of tomato fruits and not because of the tomatine, as we did not have the same increase either with pure tomatine (P) or with in vitro digested tomatine (A).

Transport of nutrients is an important function of intestinal cell. To study effect of tomatine on this function SGLT1, a sugar transporter, and PAT1, an amino acid transporter, were analysed. Exposure of the P, A, and T samples resulted in a reduced expression. Pure tomatine (P) influenced the genes more than the in vitro digested tomatine (A) and in vitro digested tomatine spiked to tomato (T); also in this case the different influence could be due to the digestion of the glycoalkaloid and/or the presence of tomato substrate.

The intestinal epithelium also acts as an integral component of the mucosal immune system producing different types of cytokines capable of initiating, sustaining and modulating the inflammatory response against injury, microbial invasions and other agonists [32,33] and this function can be influenced by dietary factors [34]. Our gene expression analysis demonstrated an influence of tomato glycoalkaloid (undigested and digested) on cytokines-mediated signaling. In particular, thymic stromal lymphopoietin (TSLP) gene expression was down regulated in all cases with no marked differences between pure tomatine (P), in vitro digested tomatine (A) and in vitro digested tomatine spiked to tomatoes (T). The expression of TSLP gene has been found induced in inflammatory bowel diseases ulcerative colitis (UC), Crohn’s disease (CD), during allergic inflammatory processes [18,35,36] and modulations or restoration of physiological amounts of TSLP have been proposed as therapeutic treatment [37]. The fact that tomatine could modulate the TSLP gene expression suggests a possible application of tomato as immunomodulating food, as also have been found for glucocorticoids [38]. Interestingly, the aglycon component of tomatine is chemically similar to the glucocorticoidal structure of steroidal hormones precursors. The expression of gene intereukin1-1β (IL-1β), a potent molecule of the innate immune system able to enhance and maintain the pro-inflammatory response [39], was induced by tomatine after 4 h exposure and even more induced when combined with digest (A) or tomato (T). It was also evident that intereukin1-8 (IL-8) transcription, a gene involved in pro-inflammatory immune response, showed to be reduced for all treatments (P, A and T) compared to media control after one hour of incubation. However, a significant up regulation of transcription levels occur after 4 h of exposure to 20 and 60 µg/mL of in vitro digested tomatine (A) and in vitro digested tomatine spiked to tomato (T). Tumor necrosis factor-α (TNF-α) is a gene involved in the amplification of inflammatory response by activating neutrophils, mononuclear phagocytes, and other cell types such as eosinophils [40,41,42]. The expression of TNF-α was up-regulated by pure tomatine (P), in vitro digested tomatine spiked to tomato (T) and in vitro digested tomatine (A) after 1 h and 4 h of exposure. These results on regulation of tomatine of immune related genes are in line with other studies that identified tomatine to have anti-inflammatory effects in rats [14,43] and to be a highly effective immunostimulator used as vaccine adjuvant in mice [14,15,44].

## 3. Materials and Methods

### 3.1. Cell Culture

Human colon cell line Caco-2, obtained from the American Type Culture Collection (ATCC) (Rockville, MD, USA), were grown on ThinCert translucent transwells (31.2 mm^2^, 0.4 µm pores, 1 × 10^8^ pores cm^−1^, Greiner Bio-one (Greiner Bio-one, Alphen aan den Rijn, The Netherlands). Cells were seeded at a density of 1.3 × 10^6^ cells mL^−1^ and grown for 21 days at 5% CO_2_, 37 °C, using Dubelco’s modified Eagle’s medium (DMEM; Gibco-Invitrogen, Bleiswijk, The Netherlands) supplemented with heat-inactivated (45 min, 56 °C) and 10% *v*/*v* fetal bovine serum (FBS; Hyclone erBio, Etten-Leur, The Netherlands). 21 days culturing of Caco-2 cells were chosen as these resemble the small intestinal enterocytes which are the first intestinal cells encountering food [43,44,45,46,47,48]. Medium was replaced three times per week. Cells were used at passage numbers from 25 to 42.

### 3.2. Materials-Chemical

A commercially available tomatine standard (a 2:1 mixture of α-tomatine and dehydrotomatine) was purchased from Santa Cruz Biotechnology, Dallas, TX, USA. 100 µg/µL tomatine stock was prepared solving standard in filtered dimethyl sulfoxide (DMSO). Eleven concentrations of pure tomatine standard (P) 0.2, 2, 5, 10, 15, 20, 40, 60, 80, 100 and 200 µg/mL, were prepared diluting the DMSO-tomatine stock with DMEM medium, adjusting the amount of DMSO per each concentration sample to expose the intestinal cells to similar experimental conditions. However, DMSO final concentrations were always less than 0.3% that is considered a tolerant concentration by cells [49]. Control cells were treated with DMEM + DMSO without tomatine (P_c_) (Table 1).

### 3.3. In Vitro Digestion

We prepared in vitro digestion of pure tomatine standard (A), tomato red fruits spiked with pure tomatine standard (T), in vitro digestion control without tomatine (A_c_), and in vitro digested tomato red fruits control without tomatine (T_c_) (Table 1). Red cherry tomatoes were purchased from a local supermarket. The in vitro digestion was adapted from previously described procedures [50]. Briefly, 15 gr of fresh tomatoes were homogenized in saline solution (NaCl 8.5 g/L). Subsequently, 20 gr of saline solution, or 20 gr of homogenized tomato were spiked with 0.2, 2, 20 or 60 μg/mL tomatine and set at pH 2, supplemented with porcine pepsin and incubated for 30 min at 37 °C. Successively, porcine pancreatin, sodium taurocholate and sodium glycodeoxycholate were added, pH was set at 6.5, the headspace was flushed with nitrogen and the samples were incubated for 30 min at 37 °C. After incubation time, pH was set at 7.5, the weight adjusted to 30 gr with saline solution and the samples centrifuged for 30 min at 3000× *g* at 4 °C. The supernatant was transferred to a new tube, flushed with nitrogen and stored at −80 °C. In vitro digested samples were filtered (0.2 µm) and diluted 1:3 with DMEM + 10% FBS before use.

### 3.4. HPLC Analysis

The final concentration of tomatine after in vitro digestion was determined by HPLC based on the chromatographic method described by [51], with slight modification. Briefly, in vitro digested samples were centrifuged at 10,000× *g* for 2 min. The supernatant was collected and concentrated by freezer-dry. The residue was suspended in 80:20 solution of methanol and 20 mM KH_2_PO_4_ (pH 3.0) and used for HPLC. HPLC analyses were performed using W6000 Waters System (Waters Corporation, Milford, MA, USA) with UV detector (Waters 2487) set at 208 nm, on an Inertsil ODS-2 column (250 mm × 4.6 mm i.d.; 5 µM particle diameter) (Hichrom, Theale, UK). The mobile phases were acetonitrile (A) and 20 mM KH_2_PO_4_ (B) pH 3.0 (elution program: 20%, 20%, 30%, 40%, 100%, 100%, 20%, 20% acetonitrile at times 0, 4, 15, 30, 31, 36, 37, 40 min), flow rate 1 mL/min. Analysis of each sample was performed in triplicate and the concentration of α-tomatine and dehydrotomatine were determined by comparing integrated chromatographic peak area from samples to peak area of known amount of tomatine standard.

The amount of tomatine detected in the samples was compared to the known amount of tomatine added before the in vitro digestion procedures. Recovery of tomatine was calculated as follows: (concentration of detected glycoalkloid after in vitro digestion)/(concentration of added glycoalkaloids before in vitro digestion) × 100.

### 3.5. Transepithelial Electrical Resistance (TEER) Assay

Transepithelial electrical resistance (TEER) was assayed using a MilliCell-ERS-meter (Millipore, Molsheim, France). TEER was measured before and after 1, 2, 3, 4 and 24 h of exposure of Caco-2 cells to each sample (P, P_c_, A, A_c_, T and T_c_). Inserts with Caco-2 cells showing TEER values < 800 Ω·cm^2^ before starting the experiments were excluded. All measurements were carried out at 37 °C in order to reduce the influence of temperature changes on cells and, then, on TEER values. Three biological replicates were used.

### 3.6. RNA Extraction and Retro-Transcription

RNA extraction and retro-transcription were performed as described by [52]. In brief, TriZol reagent (Invitrogen) was used to extract RNA from Caco-2 cells. Moreover, DNaseI (Sigma-Aldrich, St. Louis, MO, USA), RNeasy (Qiagen, Venlo, The Netherlands) and iScript (BioRad, Veenendaal, The Netherlands) kits were used to clean-up and retro-transcribe RNA. The quality of RNA samples was verified by electrophoresis on 1.2% agarose gels, and RNA concentrations were calculated spectrophotometrically (ND-1000, NanoDrop Technology, Wilmington, DE, USA).

### 3.7. qPCR Analysis

Primers for qPCR were chosen from those available in PrimerBank http://pga.mgh.harvard.edu/primerbank/ [53] for all genes except GAPDH and OCLN-2. GAPDH and OCLN-2 primer sequences were used as described by Vreeburg et al. [54] (Table 2).

Primers were purchased from Biolegio (Nijmegen, The Netherlands) and validated to optimize the qPCR conditions. qPCR was performed on C1000 Thermal Cycler (BioRad, Hercules, CA, USA).

5 μL of cDNA were added to 15 μL of a qPCR mix containing IQ™ SYBR Green Supermix (BioRad), and 100 or 400 nM of each primer. In each run, a negative control was included. Thermal cycling conditions were designed as follows: initial denaturation at 95 °C for 90 s, followed by 40 cycles at 95 °C for 10 s, 58 °C for 10 s, 72 °C for 15 s, and finally elongation temperature of 72 °C for 2 min. Glyceraldehyde-3-phosphate dehydrogenase (GAPDH) was chosen as housekeeping gene [49]. The data were analyzed calculating the relative gene expression as 2^−ΔΔ*C*^_T_ [55]. We used DMEM + DMSO control without tomatine (P_c_) as internal control for all sample in order to compare the relative gene expressions. qPCRs were performed twice for each sample of cDNA.

### 3.8. Statistical Analysis

The results were expressed as mean ± standard deviation (S.D.) of two technical repetitions. All experiments were performed in three independent biological replicates. Data were analyzed by One-way analysis of variance (ANOVA) comparing each treatment with control. *p* value < 0.05 was considered as statistically significant. For the gene expression analysis, we considered fold change criterion >1.5. All statistical analysis were performed using the GeneMaths XT software (version 2.12, Applied-Maths, Austin, TX, USA).

## 4. Conclusions

This work shows that high concentrations of tomatine can affect intestinal function as shown by exposing Caco-2 cells to different tomatine samples. In vitro digestion and tomato matrix only minimally modulate the effects induced by tomatine, probably due to a reduced stability or bioavailability. Concentrations of tomatine <20 µg/mL, both undigested and in vitro digested, might be considered safe for Caco-2 monolayers although it is difficult to extrapolate this to an in vivo situation as also cell line background and growing conditions influence the sensitivity to exposures. These supposed safe concentrations did not induce cell death or damage the monolayer integrity. Based on the gene expression analysis, we found that tomatine did not induce cell death or alter the cell cycle and no misregulation of tight junction-related genes, cholesterol/sterol biosynthesis, lipid metabolism, glucose and amino acid uptake was observed. However, we found that tomatine affects cytokine-mediated signaling genes, suggesting that tomatine might have immunomodulatory properties. To conclude, we suggest that tomatine could act as a hormetic compound that can induce beneficial or toxic effects whether used in low or high dose. Further research is needed to investigate the full safety profile of tomatine and tomatine-containing products and their potential beneficial immune supportive activity.

## Figures and Tables

**Table 1:** Samples used in this work.

Sample Name	Description
P	DMSO-pure tomatine standard
P_c_	DMESO
A	in vitro digested pure tomatine standard
A_c_	in vitro digested buffers
T	in vitro digested tomato red fruit spiked with pure tomatine standard
T_c_	in vitro digested tomato red fruits

**Table 2:** Sequences of primers used for qPCR.

Gene Name	Primer Name	Primer Sequences 5′-3′	Amplicon Lenght (bp)	Gene ID
Caspase 3	Caspase3	Fw:GAGTGCTCGCAGCTCATACCT	81	NM_004346.3
		Rev.:CCTCACGGCCTGGGATTT		
Claudin 4	F_CLDN4	Fw:TTGTCACCTCGCAGACCATC	92	NM_001305.3
		Rev:CAGCGAGTCGTACACCTTG		
Interleukin 8	F_IL8	Fw:CTGATTTCTGCAGCTCTGTG	98	NM_000584.2
		Rev:GGGTGGAAAGGTTTGGAGTATG		
Interleukin 1 beta	F_IL1B	Fw:GTGGCAATGAGGATGACTTGTTC	124	GI:27894305
		Rev:TAGTGGTGGTCGGAGATTCGTA		
Glyceraldehyde-3-phosphate dehydrogenase	F_GAPDH	Fw:TGCACCACCAACTGCTTAGC	87	NM_02046
		Rev:GGCATGGACTGTGGTCATGAG		
Tumor Necrosis Factor alpha	F_TNFa	Fw:CTGCTGCACTTTGGAGTGAT	93	NM_000594
		Rev:AGATGATCTGACTGCCTGGG		
Thymic stromal lymphopoietin	F_TSLP	Fw:TCGGCCACATTGCCTTAC	127	AY037115.1, GI:14594701
		Rev:ATAGCCTGGGCACCAGATAG		
Sodium Glucose Transporter 1	F_SGLT1	Fw:GTGCAAGTCGAGGGACCATT	114	AL109659
		Rev:GGCCGATGAACAAGCCACT		
Proton-couple aminoacid Transporter	F_PAT1	Fw:ACCTACGCACTCCAGTTCTAC	91	NM_078483
		Rev:GGTCCACCACTAACTCACAGT		
Low density lipoprotein receptor	F_LDLR	Fw:CGACAGATGCGAAAGAAACGA	142	NM_000527
		Rev:CCCGGATTTGCAGGTGACA		
Proliferating cell nuclear antigen	F_PCNA	Fw:CCTGCTGGGATATTAGCTCCA	109	NM_002592
		Rev:CAGCGGTAGGTGTCGAAGC		
Bcl-2 modifying factor	F_BMF	Fw:TTTATGGCAATGCTGGCTATCG	115	NM_033503
		Rev:GCAATCTGTACCTCTGCTTGATG		
Kluppel-like factor 6	F_KLF6	Fw:TTCTCCCACGGCCAAGTTTAC	139	NM_001160124
		Rev:CACGCAACCCCACAGTTGA		
Nuclear receptor subfamily 2, group F,	F_NR2F2	Fw:TCATGGGTATCGAGAACATTTGC	151	NM_001145156
		Rev:TTCAACACAAACAGCTCGCTC		
Occludin 2	F_OCLN2	Fw:CCCATCTGACTATGTGGAAAGA	77	NM_002538
		Rev:AAAACCGCTTGTCATTCACTTTG		
Cyclin-dependent kinase inhibitor 1A	F_CDKN1A	Fw:TGTCCGTCAGAACCCATGC	139	NM_078467
		Rev:AAAGTCGAAGTTCCATCGCTC		
